# Assessing and Harnessing the Power of Intergenerational Interactions in Mindfulness-Based Treatment Groups

**DOI:** 10.1093/geroni/igaa057.3056

**Published:** 2020-12-16

**Authors:** Deanna Dragan, Andrea Newman, Calia Torres, Keisha Carden, Sarah Letang, Danielle McDuffie, Andrew Bontemps, Rebecca Allen

**Affiliations:** 1 The University of Alabama, Tuscaloosa, Alabama, United States; 2 The University of Alabama, tuscaloosa, Alabama, United States; 3 University of Alabama, Tuscaloosa, Alabama, United States; 4 Alabama Research Institute on Aging, Tuscaloosa, Alabama, United States

## Abstract

Mindfulness-based interventions (MBIs) in individual and group formats, have been shown to be effective for a variety of psychological disorders. Due to the promising evidence supporting the wide applicability of mindfulness skills, graduate student therapists were trained to deliver groups that attracted diverse individuals across the lifespan. In these groups, therapists noted how intergenerational dynamics facilitated group cohesion and allowed for increased normalization of common challenges related to practicing mindfulness skills. Therapists’ prior training on cohort differences and treatment recommendations for older adults served as an important foundation to navigating these group interactions. Barriers to simultaneously collecting data and delivering intervention components were noted by the student therapists. Future research and therapist training gaps in knowledge related to effectively facilitating intergenerational groups were identified.

